# New insights into ice growth and melting modifications by antifreeze proteins

**DOI:** 10.1098/rsif.2012.0388

**Published:** 2012-07-11

**Authors:** Maya Bar-Dolev, Yeliz Celik, J. S. Wettlaufer, Peter L. Davies, Ido Braslavsky

**Affiliations:** 1 The Robert H. Smith Faculty of Agriculture, Food and Environment, The Hebrew University of Jerusalem, Rehovot, Israel; 2 Department of Physics and Astronomy, Ohio University, Athens, OH, USA; 3 Yale University, New Haven, CT 06520-8109, USA; 4 NORDITA, Roslagstullsbacken 23, 10691 Stockholm, Sweden; 5 Department of Biomedical and Molecular Sciences, Queen's University, Kingston, Ontario, Canada

**Keywords:** ice-binding proteins, melting shapes, ice-structuring proteins, crystal growth, antifreeze proteins, hyperactive antifreeze proteins

## Abstract

Antifreeze proteins (AFPs) evolved in many organisms, allowing them to survive in cold climates by controlling ice crystal growth. The specific interactions of AFPs with ice determine their potential applications in agriculture, food preservation and medicine. AFPs control the shapes of ice crystals in a manner characteristic of the particular AFP type. Moderately active AFPs cause the formation of elongated bipyramidal crystals, often with seemingly defined facets, while hyperactive AFPs produce more varied crystal shapes. These different morphologies are generally considered to be growth shapes. In a series of bright light and fluorescent microscopy observations of ice crystals in solutions containing different AFPs, we show that crystal shaping also occurs during melting. In particular, the characteristic ice shapes observed in solutions of most hyperactive AFPs are formed during melting. We relate these findings to the affinities of the hyperactive AFPs for the basal plane of ice. Our results demonstrate the relation between basal plane affinity and hyperactivity and show a clear difference in the ice-shaping mechanisms of most moderate and hyperactive AFPs. This study provides key aspects associated with the identification of hyperactive AFPs.

## 1. Introduction

The adaptation of organisms to cold environments has led to the evolution of a remarkable group of proteins that facilitate survival at sub-zero temperatures. These proteins have the ability to bind to ice crystals, and are therefore named ice-binding proteins (IBPs) [1] or ice-structuring proteins [2]. A subset of the IBPs includes antifreeze proteins (AFPs) and glycoproteins (AFGPs), which inhibit ice formation and growth. These proteins are expressed in a variety of freeze-avoidant organisms and contribute to their resistance to freezing [3–5]. Other IBPs allow freeze-tolerant organisms to survive in sub-freezing environments by inhibiting the recrystallization of ice [6]. These activities prevent formation of large intracellular ice crystals, which have lethal effects on the organism [4,7]. In addition, ice nucleators are found in pathogenic bacteria that use them to penetrate plant cells [8,9], and in airborne micro-organisms that have a significant role in cloud seeding [10].

By adsorption to a particular set of ice planes or sites, AFPs inhibit crystal growth normal to the bound surfaces. The crystals continue to grow on unprotected planes, if such exist, and those planes are minimized until they eventually vanish and growth ceases in all directions [11]. The ability to arrest crystal growth results in depression of the freezing temperature (*T*_f_) below the equilibrium melting temperature (*T*_m_), a non-colligative phenomenon termed thermal hysteresis (TH) [12] that is widely used to detect, define and quantify the activity of AFPs [13]. Ice crystals protected by AFPs do not expand within the range of the hysteresis gap. But when the temperature is dropped below *T*_f_, the bound proteins are insufficient to contain the crystal, and sudden, fast ice-growth occurs.

Since the first observation of AFGPs in the serum of Antarctic fishes [14], many AFPs have been identified and characterized in a variety of species, diverging remarkably in their sequences, structures and TH activities [4,15–17]. On the basis of differences in TH activity, there are two sub-groups of AFPs: the moderately active AFPs with TH activities of approximately 0.5–1°C at millimolar concentrations of purified proteins, and the hyperactive AFPs (hypAFPs), which depress the freezing point by several degrees at much lower concentrations [13,18]. The three-dimensional structures of two hypAFPs from insects: sbwAFP from spruce budworm [19] and *Tm*AFP from *Tenebrio molitor* [20], along with an Antarctic bacterial AFP, *Mp*AFP [21], and a model of the hypAFP of an inchworm [22] reveal that in spite of the independent evolutionary roots they all have flat ice-binding faces with one or more arrays of threonine side chains juxtaposed towards the solvent. The hyperactive AFP from the snow flea (sfAFP) also has a flat, two-dimensional, hydrophobic ice-binding face, although without threonine residues [23]. Recently, some of the moderately-active AFPs have emerged as a new subset of IBPs [24]. This subgroup includes proteins from plants that are associated with freeze tolerance rather than with freeze resistance. These proteins have generally lower TH activity relative to moderate fish AFPs but they are more effective in inhibiting the recrystallization of ice [24].

Directly connected with ice growth arrest is the ability of AFPs to modify ice growth habits. Adsorption to particular ice planes results in distinct ice shapes characteristic of the type of AFP. For instance, ice in the presence of type I, II or III AFPs forms bipyramidal shapes with elongated *c*-axes that are similar but distinguishable [25]. The hyperactive *Tm*AFP produces ice crystals reminiscent of the shape of lemons, or ellipsoids of revolution [26]. Specific ice morphologies and growth modifications appear even when TH activity is too low to detect, as seen with low AFP concentrations [27] or AFP analogues with reduced activities [27–32]. A systematic study of the growth habits of ice in the presence of various moderate and hypAFPs showed that a gross difference between these two subgroups is manifested in the ice growth habits at temperatures below *T*_f_ [13]. The bipyramidal ice crystals obtained with moderate AFPs grow rapidly (‘bursting’) along the *c*-axis, suggesting low or no binding to the basal planes. On the other hand, ice in solutions of hypAFPs ‘burst’ normal to the *c*-axis, and any growth by addition of water molecules to the basal planes is considerably slower than to the prism planes, suggesting an affinity of the AFPs to the basal planes [13]. Differences between the basal plane affinities of AFPs representative of the hyperactive (sbwAFP) and moderate (eel pout type III) AFP subgroups was also demonstrated by fluorescent spectroscopy [33]. In these experiments, fluorescently labelled sbwAFP was shown to accumulate on the basal plane while the eel pout AFP did not. Basal plane affinity has also been demonstrated for sfAFP labelled with green fluorescent protein (GFP) using confocal microscopy [34], and in the present study for GFP-labelled *Tm*AFP.

Here we report the differential effects of AFPs on the growth and melting habits of ice. We include in this study the IBP from ryegrass, *Lolium perenne* (*Lp*IBP) [35], which is the first representative of the expanding group of IBPs that are associated with freeze tolerance rather than with freeze resistance. We demonstrate that the characteristic shapes of ice crystals in the presence of hypAFPs are obtained during melting and stay intact in the hysteresis temperature gap. On the contrary, the bipyramidal shapes observed in moderate AFP solutions are growth shapes. We suggest that the melting processes in the presence of hypAFPs are characteristic of ice basal plane affinity, in accordance with the burst patterns. Our systematic study shows that basal plane affinity is manifested by the effects of AFPs not only on ice growth but also on melting. Ice shaping is shown to be an excellent property for distinguishing between hyperactive and moderate AFPs. We also show that the ryegrass *Lp*IBP has different shaping properties than other moderate or hyperactive AFPs and propose an explanation for its behavior. On the basis of these findings, the definition of hyperactivity can be broadened.

## 2. Material and methods

### 2.1. Protein preparation and purification

The proteins used in this study and listed below were either prepared by recombinant methods or were directly purified from the organism. Apart from the *Tm*AFP constructs and the AFGPs, all proteins were prepared and/or purified in the Davies laboratory at Queen's University, Ontario, Canada. All proteins were dissolved in 20–50 mM ammonium bicarbonate, pH ∼ 8.

Moderate AFPs:
— Type I AFP from *Pseudopleuronectes americanus* (winter flounder), isoform HPLC6. The peptide was prepared by solid-phase peptide synthesis that included the C-terminal amidation of Arg37, and was purified by reversed-phase HPLC [36].
— Recombinant type II AFP from *Hemitripterus americanus* (sea raven). The protein was expressed with a His-tag and a flanking tobacco etch virus (TEV) cleavage site [37] in *Drosophila melongaster* S2 cells. After Ni-NTA affinity chromatography, the His-tag was removed by TEV protease digestion and the AFP was recovered by ice-affinity purification [38].
— Type III AFP from *Macrozoarces americanus* (ocean pout), isoform HPLC12. The protein was expressed in *Escherichia coli* and purified and refolded from inclusion bodies as previously described [39].
— Antifreeze AFGPs. The protein sample was a gift from Dr Garth Fletcher. The sample contained primarily the 2650 Da isoform consisting of four glycotripeptide repeats. It was extracted from the blood plasma of rock cod (*Gadus ogac*) [40].
— *Lp* IBP. The protein was expressed with a His-tag in *E. coli* and purified by heat treatment followed by ice-affinity purification [41] and Ni-NTA affinity chromatography, as previously described [42].

Hyperactive AFPs:
— AFP from the beetle *T. molitor*, isoform 4–9 (*Tm*AFP). We used both a GFP fusion of the protein (GFP-*Tm*AFP) and an untagged version [43]. Both forms were expressed with a His-tag in *E. coli* and purified on a Ni-NTA affinity column. The GFP-*Tm*AFP was further purified by ice affinity [41]. The untagged protein was obtained by Ni-NTA affinity purification of the MBP-His-TEV-*Tm*AFP fusion followed by digestion with TEV protease [37], a second cycle of Ni-NTA affinity chromatography, and reversed-phase HPLC [43].
— AFP from *Choristoneura fumiferana* (spruce budworm), linked to GFP (GFP-sbwAFP). The fusion protein was expressed in *E. coli* and recovered from the insoluble fraction [44] by refolding and ice-affinity purification [41], as previously described [33].
— AFP from the Antarctic bacterium *Marinomonas primoryensis*, fused to GFP (GFP-*Mp*AFP). The calcium-dependent AFP domain fused to GFP was expressed with a His-tag in *E. coli* and purified by Ni-NTA affinity chromatography, as described [21].
— AFP from *Hypogastrura harveyi* (snow flea), fused to GFP (GFP-sfAFP). The fusion protein was expressed with a His-tag in *E. coli* and purified by two cycles of adsorption to ice [41], as previously described [34].

### 2.2. Ice crystal morphology observations

The morphologies of ice crystals during growth and melting were monitored using a homemade nanolitre osmometer, as previously described [45]. Briefly, a drop of approximately 100 µm diameter (approx. 0.5 nl) protein solution was injected into an oil-filled sample-well placed on a custom-built temperature-controlled stage and observed under a microscope. The system is controlled using a LabVIEW interface developed mainly by I.B., and the temperature close to the sample can be determined with 0.002°C precision. The stage was cooled until the drop was nucleated (usually between −27 and −35°C) and frozen, and was then warmed slowly to melt the sample such that a single ice crystal was left. This crystal was then warmed and cooled to any desired temperature several times to inspect the shaping habits. Melting experiments were regularly done at temperatures ranging from *T*_m_ to 0.05°C above it. Each experiment was repeated at least 10 times.

### 2.3. Fluorescent imaging of green fluorescent protein labelled *T. molitor* antifreeze protein

We used confocal microscopes (Zeiss LSM 510, and Nikon C1) to image ice crystals in GFP-conjugated *Tm*AFP solutions. The confocal microscope is equipped with 488 and 633 nm illumination lines and filters for the detection of GFP and Cyanine 5 (Cy5). The experimental cell used in this apparatus is the same as the one used in the nanolitre osmometer [45] but with a different sample holder. The sample holder in the present work was a copper plate with holes of either 125 µm or 1 mm in diameter, to reduce the temperature gradients. Samples of approximately 4 µl were sandwiched in between two glass coverslips and sealed with a non-cured silicone elastomer; polydimethylsiloxane (Slygard 184, Dow Corning Corp., Midland, MI, USA). The sandwiched samples were placed on the metal holder that was placed in our temperature controlled cell. In order to observe the samples inside this apparatus, we used long working-distance air objectives such as the Nikon LU Plan ELWD 50x/0.55 (WD = 10.1 mm). Samples contained a mixture of GFP-*Tm*AFP and Cy5-dUTP (PerkinElmer) as a free dye. The Cy5 dye was previously shown to have no interaction with ice [33,45] and its addition allows efficient background subtraction. To image the signal coming from the surface, we subtracted the image of non-conjugated Cy5 dye from the GFP-*Tm*AFP image, as explained in detail by Pertaya *et al.* [45].

## 3. Results

### 3.1. Moderate antifreeze proteins induce bipyramidal ice crystals during growth in the hysteresis gap

**Figure 1.**
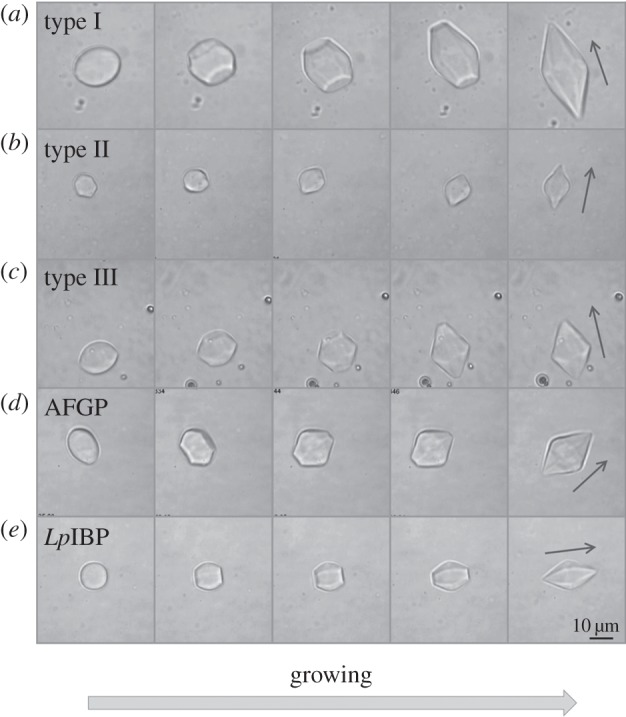
Ice growth patterns at temperatures within the TH gap in solutions of moderately active AFPs, viewed normal to the *c*-axis. Snapshots were taken during ice growth at temperatures starting from *T*_m_ (frame 1) to 0.01–0.1°C below it (frames 2–5). (*a*) Type I AFP, (*b*) type II AFP (120 µM), (*c*) type III AFP (20 µM), (*d*) AFGPs (500 µM) and (*e*) IBP from ryegrass (*Lp*IBP) (50 µM). The arrow denotes the direction of the *c*-axis. The time lapse between images 1 and 5 in (*a*–*e*) is 2 min, 3.5 min, 2 min, 40 s and 4 min, respectively.

**Figure 2.**
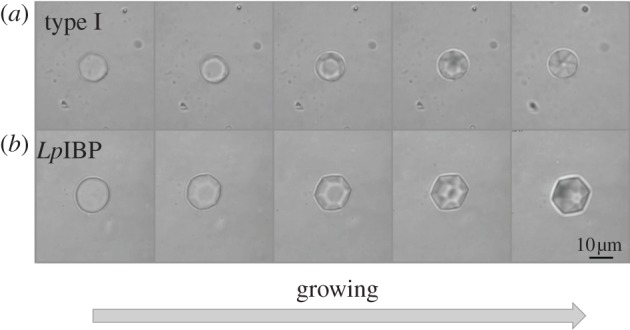
Ice growth patterns at temperatures within the TH gap in the presence of moderately active AFPs, viewed along the *c*-axis. Snapshots were taken during ice growth at temperatures starting from *T*_m_ (frame 1) to 0.01–0.08°C below it (frames 2–5). (*a*) Type I AFP and (*b*) *Lp*IBP (50 µM). The time lapse between frame 1 and 5 is 35 s and 4 min for (*a*) and (*b*), respectively.

At temperatures below *T*_m_ but within the TH gap, crystals in all solutions containing moderate AFPs tested here developed facets and edges that readily turned into truncated bipyramids with hexagonal symmetry (figures 1 and 2). These truncated bipyramids then grew in the direction of the *c*-axis, which is determined according to its twofold axial symmetry. Growth continued until tips were formed, and the basal plane was eliminated. The rate at which the tips formed was dependent on the temperature, the protein concentration and the area of the basal plane. Crystals in concentrated protein samples kept at temperatures close to *T*_m_ were maintained for relatively long time-periods (minutes) without apparent growth of the truncated tips, but if the temperature was lowered to close to the *T*_f_, the tips were rapidly sharpened to eliminate the basal plane. For example, in solutions containing approximately 20 µM AFP type III, the tips of 10 by 15 µm crystals (length versus width) grew along the *c*-axis at a rate of approximately 1 µm min^−1^ at 0.04°C below *T*_m_, but when the temperature was lowered to 0.05°C or 0.06°C below *T*_m_ the growth rate increased to approximately 6 µm min^−1^ or approximately 27 µm min^−1^, respectively. These rates were substantially reduced as the crystal lengthened and the basal planes shrank.

Once the tips of the bipyramidal crystals have grown, they appeared to remain intact as long as the temperature was kept within the TH gap. Occasionally, some local growth was observed at temperatures within the hysteresis regime close to the freezing point. This effect was more pronounced in AFGP samples. The well-known sudden rapid growth, or ‘burst’, usually initiated from the tips of the bipyramids when the temperature exceeded the TH point. Spicular ice growth was then observed along the direction of the *c*-axis (see electronic supplementary material, figure S1), as previously noted [13]. In the case of type III AFP, the burst pattern was less sharp relative to AFGPs, type I, and II AFPs, and new bipyramid-shaped ice crystals rapidly grew around the bursting crystal (see electronic supplementary material, figure S1, panel C). In all the earlier-mentioned cases, the growth in the direction of the *c*-axis is due to effective protection of the prism and/or pyramidal planes relative to the basal planes [13]. An exception to this growth pattern is *Lp*IBP, which will be discussed in §3.4.

### 3.2. Moderate antifreeze proteins induce non-bipyramidal-shaped (eye-like) ice crystals during melting

**Figure 3.**
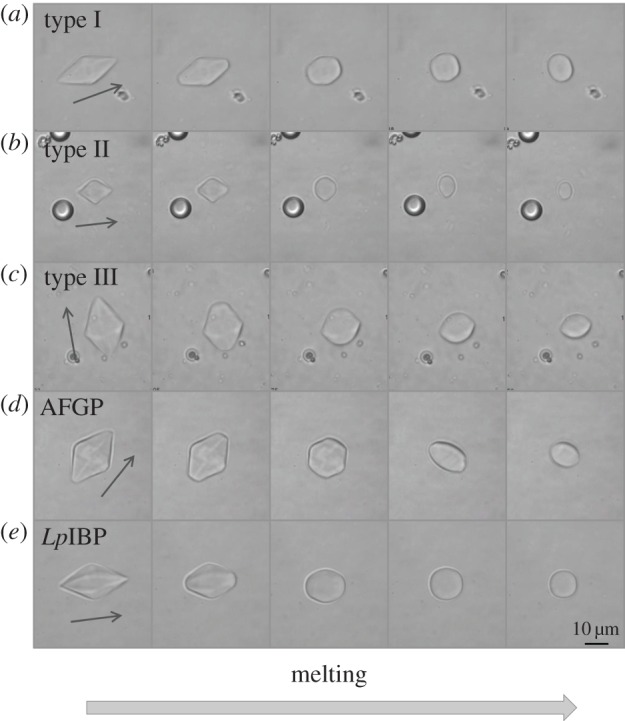
Melting of ice in solutions of moderate AFPs. The melting sequences started from bipyramidal growth shapes at temperatures within the TH gap. The temperature was then raised up to 0.05 °C above *T*_m_. The time lapse between the first and the last frame is 15–50 s. (*a*) Type I AFP, (*b*) type II AFP (120 µM), (*c*) type III AFP (20 µM), (*d*) AFGPs (500 µM) and (*e*) *Lp*IBP (50 µM). The arrow denotes the direction of the *c*-axis.

The melting pattern of the bipyramidal-shaped ice crystals obtained in moderate AFP solutions is in accordance with their formation pattern. During melting, all corners of the bipyramids disappear, and the longitudinal tips of the bipyramids shrink along the *c*-axis direction (see figure 3 and electronic supplementary material, movie S1). This melting pattern usually gives the crystals a typical shape resembling that of an eye, with the longitudinal axis being normal to the *c*-axis (figure 3, lanes 4–5). Melting in the direction of the prism planes was apparent only when the length of the *c*-axis was close to the length of the *a*-axis or even shorter (figure 3). The seemingly faceted bipyramids were recovered by lowering the temperature again (see electronic supplementary material, movie S1). The AFP-covered prism or pyramidal planes of the crystals were shielded and melting occurred at the unprotected basal planes, in accordance with the growth process.

### 3.3. The ice morphologies in the presence of insect and bacterial hyperactive antifreeze proteins are formed during melting

**Figure 4.**
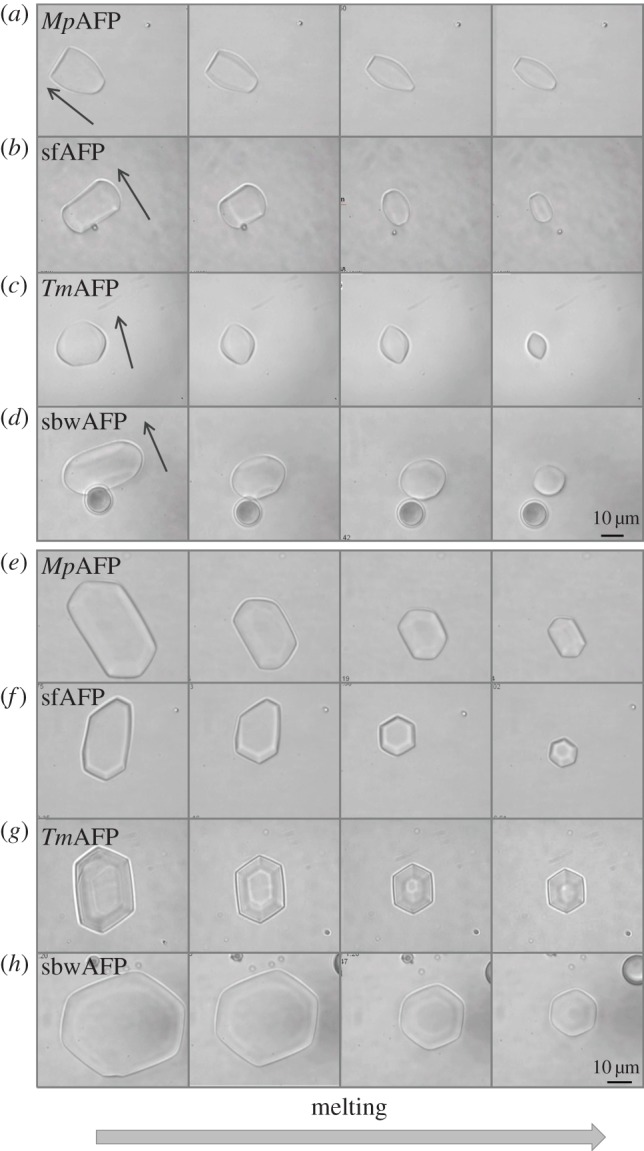
Ice melting in solutions of bacterial and insect hypAFPs. The time lapse between the first and the last frame is 15–120 s. The crystals melt in the *a*-axes direction until the basal planes are very small before melting in the direction of the *c*-axes is observed. (*a–d*) The *c*-axis is denoted by an arrow. (*e–h*) The *c*-axis is along the viewer direction. (*a*,*e*) GFP-*Mp*AFP (6 µM), (*b*,*f*) GFP-sfAFP (2 µM), (*c*,*g*) *Tm*AFP (20 µm) and (*d*,*h*) GFP-sbwAFP (8 µM).

Figure 4 shows the progression of melting ice crystals in solutions of hypAFP proteins in the concentration range of 2–10 µM. For all hypAFPs tested, it is clear that regular ice surfaces are attained during melting, and once the crystal is small enough a definitive shape is acquired. These shapes are repeatedly observed in every experiment and are so consistent that one can distinguish between the types of hypAFP solely by observing the melting process. Notably, at high protein concentrations (several tens of micromolar), faceted forms are not always obtained. Electronic supplementary material, movies S2A and S2B show that the melting is significantly faster on the prismatic planes relative to the basal planes. Macroscopically, when the temperature was close to *T*_m_, the crystals melt only from the prism planes (figure 4*a–d*, left panels and figure 4*e*–*h*) until they become elongated before melting in the direction of the basal plane can be observed (figure 4*a*–*d*, right panels). Ice crystals in the presence of *Tm*AFP melt in the prismatic direction and the basal planes shrink until they are eliminated and become only tips (see figure 4*c*,*g* and electronic supplementary material, movie S2A). This process gives rise to the previously noted lemon shape [26]. Once the lemon configuration is formed, it is maintained as long as the melting continues, from hundreds of micrometres down to few micrometres in crystal length (figure 4*c*, right panel). In GFP-*Mp*AFP and GFP-sfAFP solutions, the basal planes do not necessarily shrink down to a tip, and the shapes obtained are not as sharp and as symmetric as those observed with *Tm*AFP (and GFP-*Tm*AFP) (see figure 4*a,b*,*e*,*f* and electronic supplementary material, movie S2B). The asymmetry along the direction of the *c*-axis commonly observed in GFP-*Mp*AFP and GFP-sfAFP solutions may result from minor temperature gradients in the samples. As it is difficult to maintain the location of the sample droplet in the middle of the oil and to control the location of the crystal inside the sample, small temperature changes of a few hundredths of a degree within the sample are present. We note that despite the temperature gradients along the sample area, the temperature of the metal plate in which the sample is held is controlled with an accuracy of 0.002°C. This accuracy is the same for every position in the droplet.

With GFP-sbwAFP, the crystals usually float with their basal planes oriented normal to our line of observation and it is therefore difficult to follow melting to the direction of the basal planes. This orientation implies that the crystals are relatively flat with large, exposed basal planes. Nevertheless, formation of hexagonal shapes is clearly observed as the crystals melt (figure 4*h*). The tendency of the crystals to lie with the *c*-axis horizontally (figures 1, 3 and 4*a*–*d*) or vertically (figures 2 and 4*e*–*h*) depends on the *c* : *a* axial length ratio. Elongated crystals lie horizontally while flat crystals lie vertically due to gravity. Therefore, the typical observed melting shapes of different hypAFP consistently dictate the orientation of the crystals. In many experiments, the initial crystal has a wide basal plane before melting and therefore is oriented vertically, but as the melting progresses, the area of the basal plane diminishes, the axial ratio is inverted and the crystal turns (see electronic supplementary material, movie S2A).

**Figure 5.**
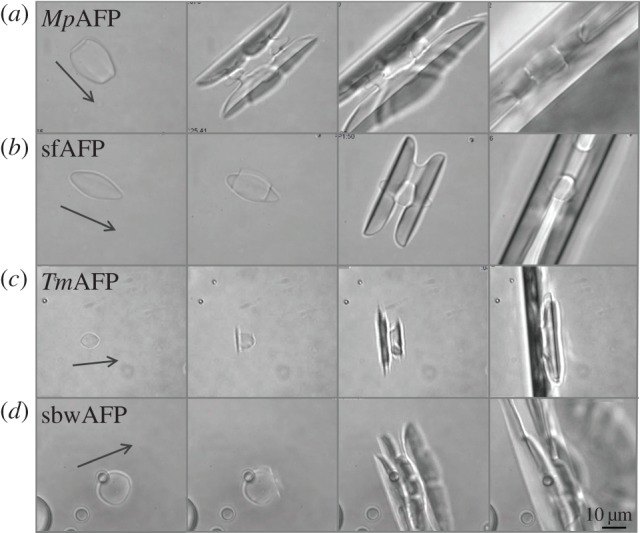
Ice growth below the freezing point (burst) in hypAFPs solutions, viewed normal to the *c*-axis. The temperature of the samples was lowered to the freezing point, and images were taken before the burst (frame 1 counted from the left) and after it (frames 2–4). (*a*) GFP-*Mp*AFP (6 µM), (*b*) GFP-sfAFP (2 µM), (*c*) *Tm*AFP (5 µM) and (*d*) GFP-sbwAFP (8 µM). (*e*) The time lapse between frame 1 and 2, 2 and 3, and between frames 3 and 4 is typically 8, 10–120 and 13–57 msec, respectively.

The ice shapes obtained during melting are stable within the TH range. When cooled below *T*_m_ the shapes do not change and the crystals do not grow, at least at the level of resolution we can detect (around a micrometre). The bottom limit of the growth arrest is the freezing point, at which sudden fast growth occurs normal to the *c*-axis. The twofold symmetry of the crystal burst is observed when it is aligned along the *c*-axis, as usually seen with GFP-*Mp*AFP, GFP-sfAFP and *Tm*AFP (figure 5). The hexagonal symmetry is apparent when the crystals are oriented normal to the *c*-axis (see electronic supplementary material, figure S2). The growth morphologies are dependent on the supercooling level and therefore on the TH activity of the sample. Ice protected by highly active protein samples is supercooled to several degrees at the lower limit of the freezing hysteresis, and the growth resembles a dendritic explosion (see electronic supplementary material, figure S2). Diluted protein solutions have low TH activity, and therefore, the degree of supercooling is small. The growth pattern in these cases is slower and smoother as expected from the growth habit of ice in water [46].

### 3.4. *Lolium perenne* ice-binding protein has mixed ice-shaping properties characteristic of moderate and hyperactive antifreeze proteins

When an ice crystal is cooled in the presence of *Lp*IBP, it grows into a hexagonal bipyramid in a manner similar to moderately active AFPs (figures 1*e* and 2*b*). Accordingly, melting of these crystals commences from the tips (figure 3*e*). Although the formation of the bipyramids and their melting pattern is as expected for a moderate AFP, the growth and melting rates seem to be slower than those of the moderate fish AFPs. A major difference with this plant IBP is the burst pattern at temperatures below *T*_f_. It commences with the flattening of the two bipyramidal tips into an expansion of the basal planes. Growth continues in a stepwise manner, mostly in the direction of the *a*-axes, while maintaining the faceted morphology of the crystal in the process (see electronic supplementary material, figure S1–E and movie S3). This pattern suggests that the protein has some affinity to both prismatic and basal planes, which is in accordance with ice hemisphere and growth experiments and the recently solved crystal structure of the protein [24].

### 3.5. Direct visualization of fluorescently tagged *T. molitor* antifreeze protein on ice crystals

**Figure 6.**
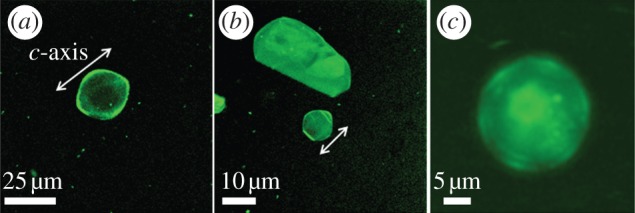
*Tenebrio molitor* AFP bind to the basal planes of ice. The fluorescently tagged AFPs visualized both on prism planes and basal plane of ice. The lemon shape was formed as the crystal size became smaller and the flat basal plane slowly melted away (*a*,*b*). The arrows show the direction of *c*-axis. In (*c*), the *c*-axis is normal to the view direction. In *b*,*c*, the melting was ceased before the basal plane shrank down to a tip.

**Figure 7.**
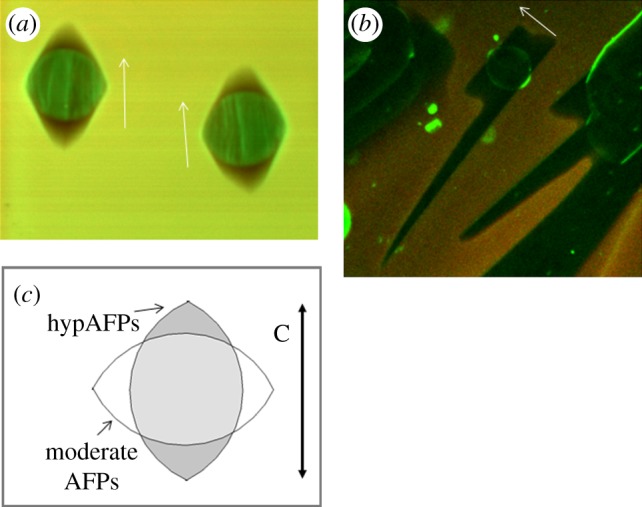
Melting versus growth shape comparison. (*a*,*b*) Fluorescent images of ice grown in AFP solutions in the presence of Cy5 for background subtraction. A frozen sample was melted until single crystals were formed, and then the temperature was dropped until growth was detected. The arrows designate the crystallographic *c*-axis. (*a*) GFP-type III AFP. The bipyramid shapes developed during growth from the eye-shaped crystal. The temperature at which the image was taken is in the TH gap. (*b*) GFP-*Tm*AFP. The crystal obtained the lemon shape during melting. The growth occurred along the basal direction when the freezing temperature was exceeded. (*c*) A diagram illustrating the crystallographic directions of the melting shapes of ice in solutions of moderate and hyperactive AFPs.

By using GFP-tagging, it is possible to directly visualize the binding of AFPs to ice. This enables us to observe where the protein is located on the ice crystal. In the presence of GFP-*Tm*AFP, the melting shape of the ice crystal becomes lemon-like as the crystals shrink in size and the basal plane becomes progressively smaller. Fluorescence confocal microscopy images confirm that hyperactive *Tm*AFP has affinity to both prismatic planes and basal plane of ice (figures 6 and 7*b*). The stronger fluorescence at the junctions between the crystal faces and edges relative to the rest of the crystal contour suggest that these junctions contain exposed areas of primary prism planes onto which the protein accumulates, as described in detail previously by Pertaya *et al*. for *sbw*AFP [33]. The fluorescence signal on the ice surfaces was also used to record the melting shapes within ice crystals that grew over their melting shapes (figure 7*a*,*b*).

## 4. Discussion

The modifications of ice growth patterns induced by AFPs have been studied for decades in order to understand the activity of AFPs and the differences between them [13,27,28,31]. Scotter *et al*. [13] have shown that when the hysteresis gap is exceeded, ice grows in the direction of the *c*-axis in the presence of moderate AFPs and normal to the *c*-axis with hypAFPs. That study established an underlying observable difference between the moderate and the hyperactive AFPs and suggested that in addition to binding to prism or pyramidal planes, hypAFPs bind to the basal planes of ice, a hypothesis that was derived primarily from the crystal structures of sbwAFP and *Tm*AFP [19,20]. Etching of a single ice crystal hemisphere grown in a solution of GFP-labelled sbwAFP revealed traces of protein on both primary prism and basal plains, giving the first experimental evidence for basal plane affinity [19]. Similar results were also obtained for sfAFP [34]. Ice etching studies of type I [47,48], type III [49] and AFGPs [50] gave no evidence of basal plane affinity by these proteins. However, the etching patterns produced by *Tm*AFP [13] are more complex and it is difficult to deduce in this method to which ice surfaces the protein adheres. The direct visualization of ice covered with GFP-*Tm*AFP presented here clearly demonstrates that the basal planes, as well as some other planes, are covered by the protein (figures 6 and 7*b*). Basal plane affinity was directly demonstrated previously for GFP-labelled sbwAFP by a series of experimental observations [33,45]. In accordance with the ice etching results, direct fluorescent studies of GFP-type III AFP demonstrated that this protein does not accumulate on the basal planes of ice [33]. We note that conjugation of AFPs to GFP does not affect the ice planes to which the proteins adhere. Partial sublimation (etching) of a single ice crystal hemisphere grown in solution of type III AFP with and without GFP conjugation [51] as well as sbwAFP [19] and GFP-conjugated sbwAFP revealed that the very same ice surfaces are bound by the two protein forms. The GFP conjugation does not affect the shaping habits of the proteins and does not reduce the TH activity. Conjugation of bulky molecules to either moderate AFPs (type III) [52] or hyperactive (*Tm*AFP [53]) actually increases the TH activity, most likely due to their increased size.

Our study of the characteristics of ice growth and melting at temperatures above *T*_f_ further supports the concept that hypAFPs protect the basal planes of ice while moderate AFPs do not. The ability of hypAFPs to cover ice from the basal plane and at least one more orientation with a significant component orthogonal to the basal plane prevents ice crystals from growing and precludes the possibility of controlling crystal shape during growth within the TH gap. This concept is shared among all hypAFPs studied here regardless of the hypothesis that different hypAFPs types bind ice from different crystallographic orientations. Moderate AFPs do not prevent ice growth in the *c* direction, and therefore growth along the *c*-axis occurs within the TH gap. Elongated bipyramidal shapes are then formed, as previously noted in TH measurements [18,54] and growth modification studies [27,31]. The dependence of the growth rate of the bipyramid tips (along the *c*-axis direction) on the *c* : *a* ratio may be related to the layer-by-layer growth mechanism of the basal planes, which requires step-nucleation events. As the surface area of the basal planes becomes smaller, the probability for a two-dimensional nucleation event is reduced, and a lower temperature or a longer time period is required to evoke such events.

It is evident that for the formation of distinct shapes, the proteins interact with ice from some crystallographic planes more than others. This concept is true for both growth and melting, although it might be more difficult to envision the fact that defined shapes can form during melting, at a time when molecules are leaving the ice front. Nevertheless, both moderate and hyperactive AFP molecules interact with ice and form unique shapes during melting. In moderate AFP solutions, the ice protection on planes parallel to or inclined to the crystallographic *c*-direction gives rise to the eye-shaped melting habit. These eye-shaped crystals have exposed unprotected planes and are therefore unstable, leading to either complete melting of the crystal or formation of the bipyramidal growth shape, if the temperature is lowered at the right moment. On the contrary, the melting shapes obtained by hypAFPs are stabilized by AFP protection from all directions, and therefore can supercool throughout the TH gap or superheat for a certain range of temperatures (approx. 0.4°C for *Mp*AFP) [55] without a change of the crystal shape.

To emphasize the differences between the growth and melting shapes, we introduced fluorescent images of crystals that contain contours of both types of shape. Ice grown from the melt in GFP-type III AFP solutions has a strong fluorescent signal inside the eye-shaped melting forms and around the bipyramidal growth shapes (figure 7*a*). The eye-shaped region in the centre of the crystals is the area of the original crystal before it grew to form the bipyramid. The strong fluorescent signal in this region is due to protein present inside, originating from the fast freezing process at approximately −20°C of the sample before it was melted to obtain single crystals. At these low temperatures, fine crystals are grown and GFP-AFP type III is accumulated typically on prismatic planes and some other planes slightly tilted from them [49]. The high fluorescence remains at the unmelted portion of the ice crystals. When these crystals were allowed to grow at temperatures within the TH gap, protein was excluded from the ice front. This area of the crystals has a correspondingly low fluorescence signal. A fluorescent layer of protein appears on the outer region of the bipyramidal crystal, where the planes to which the protein adsorbs are exposed. The observation of the strong fluorescent area inside the crystals is consistently observed in our experiments and was noted previously, although the phenomenon was not explained [33,45]. For comparison, crystals obtained in the same process in solutions of GFP-labelled sbwAFP and *Tm*AFP remain in their melting shapes and have strong fluorescent layers around them ([56]; figures 6 and 7*b*). This protein layer protects these crystals from both growth (within the TH range) and melting [55], and the crystals are therefore stable in their melting forms. Nevertheless, the interior areas of these crystals have weak fluorescent signals relative to the crystals in moderate AFP solutions, which may be due to slower absorption kinetics of the hypAFPs relative to the moderate proteins [55]. Further work is needed to quantify the protein present on the surfaces of the crystals and inside them to directly evaluate the effectiveness of ice coverage by hypAFPs relative to moderate AFPs.

In comparison with the bipyramidal growth shapes obtained with moderate AFPs within the TH gap, the growth shape of ice in hypAFP solutions is observed only when the TH gap is exceeded (see figures 5, 7*b*, and electronic supplementary material, S2). In figure 7*b*, the melting shape of the crystal after burst is clearly observed by the fluorescent layer surrounding it. The directionalities of the melting shapes of crystals in moderate AFPs solutions are compared with the melting shapes observed with hypAFPs in figure 7*c*. Owing to basal plane affinity of hypAFPs, the crystals grown in hypAFP solutions melt normal to the *c*-axis and the basal planes shrink without change of the distance between the two basal faces. This is in contrast to the melting habits in solutions of moderate AFPs. Therefore, the longitudinal axis of the melting shapes obtained with hypAFPs is the crystallographic *c*-axis, while the longitudinal direction of the melting shapes obtained in moderate AFP solutions is normal to the crystallographic *c*-axis.

The morphology of the bipyramids observed in hypAFP solutions is dependent on the particular crystallographic orientations to which the protein binds and on the kinetics of the binding. Different binding rates to the various crystallographic directions evoke differences in the melting velocities of these directions, which gives rise to a typical crystal shape. Indeed, the melting velocities of crystals in the presence of hypAFP were measured and correlations between the type of AFP to the velocity at a given superheating level were found [55]. This scenario is consistent with the observations that each protein type consistently produces characteristic, distinguished shapes in many experiments, such as the lemon shape obtained with *Tm*AFP (figure 6). In a recent work on ice growth patterns with AFGPs and AFGP analogues, the authors suggest that the modifications observed in crystal morphologies and axis ratios are related to the adsorption kinetics of the AFP to particular ice faces [28]. The adsorption rates are presumably the key factor in the determination of the shape of the crystal, whether it was obtained during growth or melting [57]. A different explanation for ice shaping may rely on different affinities to particular ice surfaces, which is reflected by different desorption rates and not only adsorption rates. This explanation is ruled out by the claim for irreversible binding of AFPs with ice [33,58,59].

The concept of shape formation during melting due to different melting velocities along the crystallographic directions has recently been supported by a theoretical simulation in three dimensions [60]. The three-dimensional simulation was an extension of a two-dimensional geometric model for crystal growth that relates the growth velocity to each direction to the final shape obtained [61]. A drum-shaped structure was allowed to ‘melt’ by applying a velocity profile with a twofold symmetry in the *z* direction and a sixfold symmetry in the orthogonal plane. To reflect the basal plane affinity of the hypAFPs, the velocity in the *z* direction was considerably smaller than in the *x*–*y* directions. This simulation resulted in a crystal with a lemon-like shape, strikingly similar to the ice shapes experimentally observed in *Tm*AFP. This result relates slow melting rate in the direction of the basal plane to the observed melting shapes. Further development of this approach is necessary to obtain other melting shapes, which may aid in explaining the differences between the melting processes of the various hypAFPs and the hyperactivity phenomenon altogether.

An explanation for the hexagonal symmetry normal to the *c*-axis of the melting crystals was previously given in the study of Pertaya *et al*. [56]. They showed that the prismatic planes protected by AFPs are not the same planes observed during melting. Prismatic-protected planes were obtained during growth in dilute solutions of GFP-labelled sbwAFP, and melting was shown to be induced at the corners of those protected planes, resulting in a new set of planes, rotated by 30° relative to the growth planes, similar to the growth-melt asymmetry found in pure ice under high pressure [62]. The newly formed planes are those observed during melting, and the junctions between these planes, where the original growth planes are exposed, are shown to be protected more efficiently by AFPs relative to the melting planes. Furthermore, it is reasonable to assume that the corners of the bipyramids expose several crystallographic planes onto which the proteins may accumulate, leading to the strong fluorescence signal at the corners relative to the edges. A similar phenomenon is observed in GFP-labelled *Tm*AFP (figure 6), but with less profound accumulation on the corners in comparison with GFP-sbwAFP.

The moderate ryegrass *Lp*IBP exhibits ice bursts perpendicular to the *c*-axis, contrary to other moderate AFPs. This finding implies that there is some affinity of this protein for the basal plane of ice, in accordance with ice hemisphere studies [24]. The recently solved structure [24] and ice-binding face [42] of this protein provides an explanation for the phenomenon of basal plane affinity despite low TH activity. The ice-binding face of *Lp*IBP is reminiscent of the orderly array of threonines observed on the ice-binding faces of several hypAFPs [19–22]. Nevertheless, this array of exposed side chains is considerably less repetitive than in the hypAFPs, and contains only a few threonines [24,42]. This composition of residues may result in the reduction of the binding rates or even binding affinities of *Lp*IBP to both prism and basal planes, yielding the low TH activity of this protein [24,35,63]. In comparison, a mutant of *Tm*AFP with four out of the 10 threonine residues constituting the ice-binding face replaced by valines had TH activity comparable to *Lp*IBP [29]. This mutant could not block ice growth completely, suggesting that it has a reduced affinity to ice. Despite its low TH activity, *Lp*IBP has high ice recrystallization inhibition and ice restructuring properties [64] relative to other AFPs. This set of properties of *Lp*IBP, and presumably other plant AFPs (or IBPs), aides in the protection of the plants from ice damage and from excessive supercooling.

The differences in the ice-shaping patterns between hypAFPs and moderate AFPs demonstrated here have significant implications for the utility of AFPs in a variety of fields such as cryopreservation and cryosurgery. For example, it was shown that although type I AFP increased the viability of red blood cells after a freeze–thaw cycle, lethal effects were observed at high concentrations of this AFP owing to extensive growth of extracellular ice [65,66]. Cellular membranes might have been damaged by the needle-like ice crystals produced by type I AFP. In fact, the use of moderately active AFPs in cryopreservation procedures might be limited by these spicular structures obtained in their presence when the hysteresis freezing point is exceeded. HypAFPs do not lead to the formation of such sharp ice crystal shapes and may therefore be better candidates for cryopreservation. Nevertheless, in cryosurgery studies, lethal effects associated with moderate AFPs have been shown to be advantageous [67]. In this case, using hypAFPs may be less effective. Thus, the ice-shaping properties of the various AFPs may be a critical factor when selecting an AFP for a particular task. In this respect, it would be interesting to examine the ice-shaping habits of other cryogenic substances and antifreeze agents such as xylomannan [68] and zirconium acetate [69] and compare them with the proteins studied here.

## 5. Conclusions

Although the ice-shaping properties of hypAFPs have been studied, they have been uniformly referred to as growth shapes, analogous to those formed by moderate AFPs. The fact that these shapes are formed during melting has been generally ignored. Our work demonstrates that the crystal shapes obtained with hypAFPs are obtained during melting and not during growth. This phenomenon is directly related to the ability of hypAFPs to protect the basal plane of ice crystals in addition to other planes. Therefore, in hypAFPs solutions, changes in ice morphology do not occur in the TH regime, and the shaping occurs during melting owing to the influence of the proteins on interfacial melting rates. In agreement with these observations, melting simulation results show that a lemon-like shape can be obtained during melting if the basal planes of the crystals are covered and melting in this direction is suppressed [60]. For moderate AFPs, which do not block the basal plane growth, the observed bipyramidal shapes are obtained during growth within the TH regime. The exception of *Lp*IBP to the generalization that relates basal plane affinity with hyperactivity and sharply divides AFPs between ‘hyperactive’ and ‘moderate’ hints that perhaps this classification needs to be broadened. The findings of this study, particularly that ice reshaping occurs during melting, may enable the construction of better models to describe the interactions of AFPs and ice.
